# Perforation in Paratyphoid B

**Published:** 1930

**Authors:** Frank Bodman, Paul Bodman

**Affiliations:** Physician, Bruce Wills Memorial Hospital; Assistant Physician, Bruce Wills Memorial Hospital


### PERFORATION IN PARATYPHOID B.

BY

Frank Bodman, M.B., M.R.C.S.,

*Physician, Bruce Wills Memorial Hospital,*

AND

Paul Bodman, M.B.,

*Assistant Physician, Bruce Wills Memorial Hospital.*

---

In view of the rarity in civil practice the following case would seem worth recording :—

J. B., aged 64, was a tall, well-built gentleman. His previous history was only remarkable for a prostatectomy performed some six years before, with a good result as far as micturition was concerned. He had suffered since the operation from one or two attacks of colic, which had suggested some temporary intestinal obstruction. In April, 1930, he developed a carbuncle ; this cleared up satisfactorily without any evidence of glycosuria.

The patient was first seen on 23rd July, when he had already had two days of slight malaise, but awoke that morning feeling ill and aching all over ; he complained of headache, thirst and shivering. The temperature in the evening was 101·2°, and next day remained at the same level, and the symptoms were unchanged. A blood-film was made in the evening, and the differential white cell count was : polymorphs 74 per cent., lymphocytes 22 per cent., hyalines 4 per cent.

On the third morning the temperature was normal, but on the fourth day it rose to 102·8° and on the sixth day to 103·8°. About the fourth day the stools, hitherto hard and infrequent, became loose, urgent, almost involuntary, occurring at first about once in twenty-four hours.

Blood was taken on the sixth day and yielded on culture an organism of the lactose fermenting group with agglutination reactions of paratyphoid B. to a high titre. By the seventh day the condition had become typically "typhoid." The patient lay supine, with little desire to move, face flushed and moist, tongue increasingly furred and dry, pulse at first disproportionately slow, though not dicrotic. Occipital headache, mild nocturnal delirium, increasing tympanites with occasional pinching pains in the hypogastrium and umbilical region. Although disinclined for conversation, he showed a remarkable mental acuteness, considering the general toxæmia.

During the second week of the illness this condition persisted without any appreciable increase in the severity of the symptoms, except for the inevitable emaciation. The stools became more frequent, two or three in twenty-four hours, greyish, watery and very offensive. The abdominal discomfort was most marked in the right iliac fossa. Rose spots were never seen in spite of careful search. Towards the end of the fourth week, as the pyrexia still continued, and the general condition of the patient was not improving, Dr. Carey Coombs was consulted. The patient was complaining of a good deal of dysuria, and there had been several slight rigors. Dr. Coombs suggested the possibility of a *B. coli* infection and a sample of urine was submitted to Dr. H. Rogers, who reported on the presence of pus cells and a very heavy growth of organisms. Typhoid bacilli were present and also a large number of abnormal *B. coli*. Sub-cultures of these *B. coli* on a fresh blood-plate failed to show any marked hæmolytic effect. A marked urobilinuria was present.

With great difficulty the patient was encouraged to increase his fluid intake, but he constantly complained of colicky abdominal pain immediately after eating or drinking. During the next week the fever continued, and the patient became more and more distended. Visible peristalsis was observed on several occasions. On the thirtieth day a few small blood-stained sloughs were noticed in the stools; there was still marked distension, but no tenderness. The intake of food was cut down, and attempts were made to relieve the pressure in the gut by carefully introducing a little normal saline per rectum and siphoning off. This afforded some relief, but on the morning of the next day, after a similar manœuvre, the patient suddenly complained of severe abdominal pain. Seen shortly afterwards in consultation with Dr. Birrell, the abdomen was distended and rigid. The temperature had fallen from

103° to 96° and the facies was hippocratic. There seemed no doubt that perforation had taken place, but the condition of the patient was so very grave that operative interference was unjustifiable. The pulse and temperature rose rapidly, and seven hours after the first symptoms of perforation the patient was dead.

There was no post-mortem examination, but the clinical picture appears to justify us in describing this as an example of intestinal perforation in paratyphoid B.

From 1902, when Buxton differentiated the paratyphoids, up to the time of the Great War, it was generally held that paratyphoid was a relatively mild illness without serious complications. Allbutt and Rolleston [1] (1905) record 160 cases without perforation ; Osler and MacCrae [2] (1908) just mention it ; Brouardet and Gilbert [3] state that perforation has never occurred ; and Ribadeau-Dumas [4] is still more emphatic : "les perforations intestinales n'existent jamais."

But the experience of the war threw a different light on paratyphoid. Torrens and Whittington [5] estimated the frequency of perforation in paratyphoid B at 1 per cent. from their war experiences. Vincent and Muratet [6] believed it to be as frequent an occurrence as in typhoid proper. Dawson and Whittington [7] published two cases of perforation in paratyphoid B. Webb-Johnson, [8] in his Hunterian Lecture, reported three cases of perforation in 799 cases of uninoculated paratyphoid B. In the discussion at the Royal Society of Medicine (1915) Robinson [9] criticized Torrens' figures as too high, but Sir William Leishman, [10] out of approximately 840 cases of paratyphoid B, gave a mortality of 1·5 per cent., mostly perforations. Rathery, [11] in his report from a French army hospital, quotes several cases of perforation, and Sir Wilmot

Herringham[12] called attention to the fact that perforation could occur in paratyphoid. Grener[13] reported a case in full in the French medical press; his case was probably typical of many, as it occurred in a case of ambulatory typhoid. The result has been that in editions of the text-books published since the war perforation in paratyphoid B is admitted as a possibility; for example, by Box[14] (1922), Goodall[15] (1922), Osler[16] (1925), Kerr[17] (1927), Manson-Bahr[18] (1929), Stitt[19] (1929).

But the infrequency in civil as compared with military practice is commented on by Woolf.[20] A prolonged search of the literature has revealed only a very few cases since the war. Cases of perforation in paratyphoid B have been reported by Woolf,[20] Süssengäth,[21] Dvorak and Lubormirsky,[22] Pop[23] and Cornils.[24]

The interest in this case depends also on the late date of perforation, the thirty-first day. I only found record of a perforation occurring at a later date (thirty-third day) in one case of Webb-Johnson's.[8] This perforation occurred in the descending colon. It is probable that in this case the perforation may also have been in the colon, as the signs of perforation came on after a saline wash-out, and there was evidence of the colonic involvement so typical of paratyphoid B in the marked tenesmus.

The *B. coli bacilluria* was another interesting point. Nabarro[25] has suggested that in intestinal infections the *B. coli* may acquire pathogenic properties and add to the complications. The *B. coli* infection of the urinary tract was, no doubt, a factor in maintaining the prolonged pyrexia. That mixed infections can occur was demonstrated by war experience, as several workers reported during the war on mixed infections of typhoid and paratyphoid, of paratyphoid and Shiga infections. The marked tympanites, together with visible peristalsis, was a significant sign in this case. It is possible that there was some intestinal obstruction, due to adhesions to the abdominal wall after the prostatectomy. The extra pressure on the wall of the gut was probably a determining factor in perforation.

The condition of this particular patient was so bad after perforation that operative interference seemed hardly justifiable. Potel and Minet [26] have, however, recorded a case in a younger subject who recovered from a typhoid perforation after simple drainage of the peritoneum. Lejars [27] has stressed the importance of hemorrhage preceding perforation, and it is interesting to note that in this case perforation was preceded forty-eight hours before by slight intestinal hemorrhage.

### REFERENCES.

1 Allbutt and Rolleston, *System of Medicine*, 1905.

2 Osler and MacCrae, *System of Medicine*, 1908.

3 Brouardet and Gilbert, *Traité de Médecine.*

4 Ribadeau-Dumas, *Journ. Med. franc.*, August, 1910.

5 Torrens and Whittington, *Brit. Med. Jour.*, 1915, 13th Nov.

6 Vincent and Muratet, *Typhoid and Paratyphoid Fevers*, 1917.

7 Dawson and Whittington, *Quart. Journ. Med.*, 1915–16, ix. 98.

8 Webb-Johnson, *Surgical Aspects of Typhoid and Paratyphoid Fevers*, London, 1919.

9 Robinson, *Proc. Roy. Soc. Med.*, 1916, ix. 27.

10 Leishman, *ibid.*, p. 17.

11 Rathery et alia, *Les fièvres paratyphoides B à l'hôpital mixte de Zuydcoote*, 1916.

12 Herringham, *Lancet*, 1915, ii. 995.

[13] Grener, *Progrès méd.*, 1913, xxix. 114.
[14] Box, *Price's Textbook of Practice of Medicine*, 1922.
[15] Goodall, *Short's Index of Prognosis*, 1922.
[16] Osler, *Principles and Practice of Medicine*, 10th Ed., 1925.
[17] Kerr, *Manual of Fevers*, 1927.
[18] Manson-Bahr, *Manson's Tropical Diseases*, 1929.
[19] Stitt, *Tropical Diseases*, 1929.
[20] Woolf, *Brit. Journ. Dis. Child.*, 1923, xx. 91.
[21] Süssengath, *Munch. med. Woch.*, 1918, lxv. 915.
[22] Dvorak and Lubormirsky, *Casop lék Ĉesk.*, 1929, lxviii. 885.
[23] Pop, *Zentrablatt f. Chir.*, 1929, lvi. 77.
[24] Cornils, *Deut. Zeitsch. f. Chir.*, 1924, clxxxvii. 422.
[25] Nabarro, *Lancet*, 1930, ii. 415.
[26] Potel and Minet, *Écho médical du Nord*, 1909, i. 58.
[27] Lejars, *Urgent Surgery*, 8th Ed., 1923, 362.

